# Statistical Evaluation of Barkhausen Noise Testing (BNT) for Ground Samples

**DOI:** 10.3390/s19214716

**Published:** 2019-10-30

**Authors:** Robert Tomkowski, Aki Sorsa, Suvi Santa-aho, Per Lundin, Minnamari Vippola

**Affiliations:** 1 Manufacturing and Metrology Systems, Department of Production Engineering, School of Industrial Engineering and Management, KTH Royal Institute of Technology, Brinellvägen 68, 114 28 Stockholm, Sweden; 2 Control Engineering, Environmental and Chemical Engineering, Faculty of Technology, University of Oulu, P.O. Box 4300, FI-90014 Oulu, Finland; aki.sorsa@oulu.fi; 3 Faculty of Engineering and Natural Sciences, Tampere University, P.O. Box 589, FI-33014 Tampere, Finland; suvi.santa-aho@tuni.fi; 4 Schlumpf Scandinavia AB, Flygfältsgatan 2D, 128 30 Skarpnäck, Sweden; per.lundin@schlumpf.se

**Keywords:** Barkhausen noise testing (BNT), uncertainty, proficiency test, ANOVA

## Abstract

Barkhausen noise testing (BNT) is a nondestructive method for investigating many properties of ferromagnetic materials. The most common application is the monitoring of grinding burns caused by introducing locally high temperatures while grinding. Other features, such as microstructure, residual stress changes, hardening depth, and so forth, can be monitored as well. Nevertheless, because BNT is a method based on a complex magnetoelectric phenomenon, it is not yet standardized. Therefore, there is a need to study the traceability and stability of the measurement method. This study aimed to carry out a statistical analysis of ferromagnetic samples after grinding processes by the use of BNT. The first part of the experiment was to grind samples in different facilities (Sweden and Finland) with similar grinding parameters, different grinding wheels, and different hardness values. The second part was to evaluate measured BNT parameters to determine significant factors affecting BNT signal value. The measurement data from the samples were divided into two different batches according to where they were manufactured. Both grinding batches contained measurement data from three different participants. The main feature for calculation was the root-mean-square (RMS) value. The first processing step was to normalize the RMS values for all the measurements. A standard analysis of variance (ANOVA) was applied for the normalized dataset. The ANOVA showed that the grinding parameters had a significant impact on the BNT signal value, while the other investigated factors (e.g., participant) were negligible. The reasons for this are discussed at the end of the paper.

## 1. Introduction

**Figure 1:**
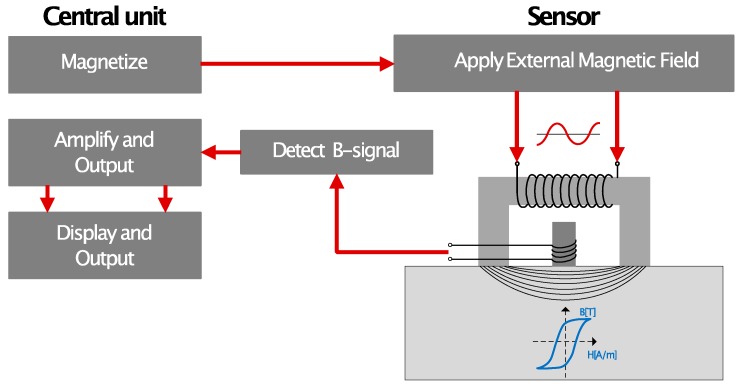
General scheme of Barkhausen test equipment.

Nondestructive testing (NDT) of machine parts’ surface integrity has grown greatly over the last 20 years. There are many methods and techniques, based on different physical effects, that can be used for NDT (Figure 1). Faster and more efficient process control leading to reduced set up time can be achieved through the introduction of proper methods and innovative concepts for nondestructive analysis and verification of product quality, especially for altering the component type in the production line [1].

One of these NDT methods is Barkhausen noise testing (BNT), which is utilized to assess changes in the surface layer of ferromagnetic materials, especially to monitor changes in their hardness and residual stresses. BNT is based on the interaction between the external magnetic field and the ferromagnetic material. The reorganization of the magnetic domains and the formation of an internal magnetic field are registered by the sensor. The magnitude of the registered signal and its parameters depend on many factors. Many of them are noncorrelated, while others share a strong correlation. One can easily demonstrate that the set of factors affecting the Barkhausen signal comprises more than 200 components, including interactions between factors (Table 1). The combination of all these factors results in the material response of external magnetization.

The basic equipment consists of a measuring device with a sensor and connecting cable to generate and measure Barkhausen noise (BN) (Figure 1).

The BN analyzing instrument requires both generating the applied magnetic field, in order to send it into the material, and picking up and presenting the BN signal generated from the material. For that purpose, BN sensors are used. There are normally two primary functions of a BN sensor. First, an external magnetizing field is applied, which penetrates the surface of the material to be analyzed. For that, a magnetic yoke with two magnetic pole pieces is required. The orientation of the magnetizing pole pieces determines the direction of the applied field and also the measurement direction of the BN signal. Second, some kind of sensor that can detect the BN signal is needed. The most common way is to use a sensing coil that can be tailored to the analyzing frequency. Alternatively, a Hall element can be utilized. There is also the possibility of using an external magnetic yoke to generate the applied magnetic field and then using a pick-up sensor for the BN signal. The available BN sensor types are often classified into two groups: surface-specific sensors (external and internal) and product-specific sensors (camshaft, crankshaft, gears, etc.). The surface-specific sensors have a broader range of application and are divided into general purpose sensors, flat sensors, outer diameter (OD) sensors, and inner diameter (ID) sensors. The product-specific sensors are oriented for specific components, such as camshafts, crankshafts, gears, or other components.

The generated BN signal needs to be picked up by a sensing coil or element, amplified, filtered, and presented. The presentation can either be in numbers on a display or as an oscilloscope signal that can be stored and further analyzed. The BN signal is analyzed by examining features computed representing, for example, Barkhausen activity and the shape and position of the BNT envelope. BNT is a stochastic phenomenon and thus only averaged properties are reproducible. The use of a high sample rate and acquisition of time-related data during a specified number of magnetizing cycles, typically 10 BN bursts, is required to obtain good averaging. The BN signal is acquired over a larger analyzing frequency spectrum, for example, 1–1500 kHz, which makes it possible to later select different or narrower bands of analysis. Typical features studied are, for instance, the root-mean-square (RMS) value, peak height, peak width, and peak position concerning the signal. Both the pulse-like noise signal and envelope of the BN burst can be analyzed. Also, the amplitude spectrum and pulse height spectrum can be studied to obtain information concerning the material properties.

A challenge with BNT is that the measured values are not reproducible and depend on the measurement arrangement. The sensor, measurement parameters, signal processing parameters, and the issues related to the measurer may influence the measurement result. In this study, an interlaboratory proficiency testing was carried out to evaluate if participant-related issues are significant. Three laboratories with similar equipment performed BN measurements for two sets of samples. The samples were ground with different grinding parameters to obtain changes in the BN response. A standard analysis of variance (ANOVA) was carried out to distinguish between the effects of grinding parameters and the measurer.

This study also evaluated how measurement uncertainty decreases as the BN measurement is repeated. For this measurement, uncertainty was computed as a function of repetitions. Two uncertainty indices were computed, the first emphasizing the average expected uncertainty, while the other considered the worst-case scenario of maximum uncertainty. This study highlights the significance of repetitions to draw valid conclusions.

## 2. Proficiency Testing

Usually, proficiency testing is carried out as one essential activity of testing laboratories and it has become a mandatory requirement for laboratory accreditation. The testing ensures that the statistical methods which are adopted are fit for the intended purposes [2]. Generally, the proficiency testing scheme is at first described according to the intended objective and purpose of the study. Then, the statistical test plan with methods is performed. The last phase is to evaluate the results from the individual test laboratories (performance evaluation).

The samples can be tested either by each laboratory by themselves with certain instructions [2] or laboratories can use a group of samples made by a certain party which is distributed to them [3]. We studied pressure vessel samples with different annealing treatments (thermal degradation) as one project partner in a round-robin BNT study.

BNT itself is affected by many factors: the equipment [4], the sensor design [5], the participant [6], the way the measurement is carried out [7], and the software used. Generally, gauge, repeatability, and reproducibility (GR&R) tests are used when studying measurement variations and their causes, which include the effects of a participant, effects of the equipment, and the way the measurements are carried out. We studied, among other things, the use of different statistical calculation tools for testing the BNT equipment’s performance (repeatability and reproducibility) in a quality check before sending BNT equipment to customers.

Fewer BNT-based round-robin studies have been carried out than X-ray-diffraction-based residual stress round-robin studies, which have been performed by, for example, [8] and [9]. Regarding BN round-robin studies, even as early as 1977, a round-robin activity study was performed measuring railroad wheels and evaluating their residual stresses with different methods, including the BN method [10]. The round-robin studies involved several research institutes, but the BNT measurements were carried out by only one research institute. The outcome of the BNT measurements was compared to the results obtained with other methods.

Takahashi’s group studied the degradation of ferromagnetic materials with BNT [11]. Their round-robin studies in the Universal Network for Magnetic NDE (Non-Destructive Evaluation) concentrated on the evaluation of the measurement technique to help the standardization procedure of magnetic BNT. However, the BN results showed considerable disagreement among the participating groups and the most likely reason for this was stated to be the differences in the measurement techniques.

The study and analysis of differently ground samples was the objective of this interlaboratory round-robin test that involved researchers from three different laboratories in Sweden and Finland. The first part of the experiment was to prepare two batches of ground samples in different facilities with similar grinding parameters. The second part of the experiment consisted of an interlaboratory round-robin comparison carried out with the magnetic Barkhausen noise method. The main tasks were (1) interlaboratory comparison and (2) evaluation of the effect of grinding on Barkhausen noise features.

## 3. Materials and Methods

In this experiment, the near-surface influence on magnetic BNT was investigated by grinding-hardened specimens of various hardness values with different abrasives and intensities.

### 3.1. Design of Experiment

We implemented a full factorial experiment design with three repetitions [12]. Three factors were chosen: hardness, abrasives, and intensities, each at two levels. That gave $k\cdot 2^{p}$ experiments, where *k* is the number of repeated experiments and *p* is the number of factors studied. This gave $3\cdot 2^{3}=24$ experiments. Furthermore, the experiments were repeated for two sets of samples, giving a total number of 48 experiments. Running the full experiment design with all possible factor combinations meant that all of the main and interaction effects could be estimated. For three factors at two levels, this meant three main effects, three two-factor effects, and one three-factor effect. This combination is described by the following model:

(1) $$Y=\alpha_{0}+\alpha_{1}X_{1}+\alpha_{2}X_{2}+\alpha_{3}X_{3}+\alpha_{12}X_{1}X_{2}+\alpha_{13}X_{1}X_{3}+\alpha_{23}X_{2}X_{3}+\alpha_{123}X_{1}X_{2}X_{3}+\epsilon$$

This model allows estimation of all α coefficients $\left\{\alpha_{0},\cdots ,\alpha_{123}\right\}$ and the analysis of significance of the terms. The design applied can be improved by adding at least three center point runs.

### 3.2. Materials

Round bar samples manufactured from 20MnCrS5+A steel (Table 2) with a diameter of 40 mm and a height of 35 mm were used in this study. The samples were carburized case hardened with oil quenching. The total hardening depth was max. 1.2 mm. In total, 56 samples were prepared and divided into two batches for further processing. Half of the carburized samples were also tempered at 180 °C for 1.5 h. After the heat treatments, a carefully planned grinding procedure was carried out for the samples.

### 3.3. Grinding Plan

**Figure 2:**
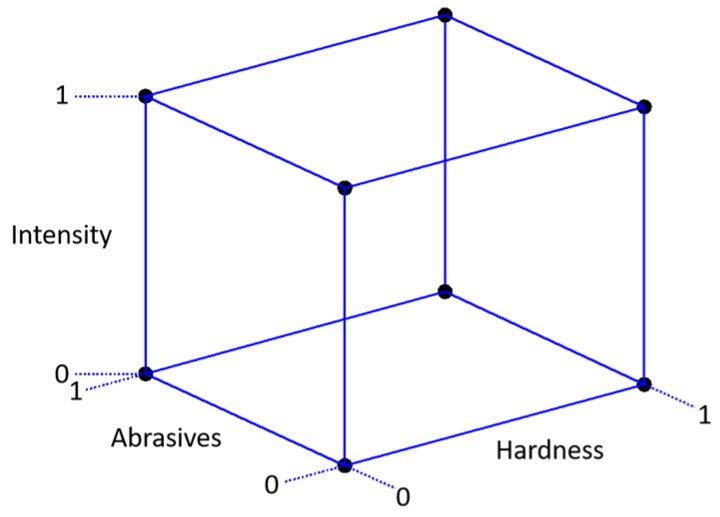
Grinding plan.

Two different grinding batches were prepared in different grinding facilities. In total, three different variables were changed in the grinding, as shown in Figure 2.

The parameters of the grinding experiment are presented in Table 3. All of the specimens were case-carburized to a hardness of 63 HRC. Half of the hardened specimens were tempered at 180 °C for 1.5 h to differentiate the hardness. In total, 48 samples were studied, 24 for each batch. 

The CBN grinding wheel grain size varied and was either B126 or B181 (Ilyich Abrasive Company, Saint Petersburg, Russia). The grinding intensity was altered by carrying out the grinding of, in total, 0.6 mm stock removal in either four or eight steps (batch #1) or in three or six steps (batch #2). The grinding wheel speed was 35 m/s, and the grinding table speeds were 8 m/min (batch #1) and 10 m/min (batch #2). The cooling fluid (water emulsion 5%) flow was 15 L/min. Four samples were ground by the normal grinding procedure, of which two were in a hardened condition and two were tempered. Normal grinding was carried out with a B126 grinding wheel with a high number of steps.

### 3.4. Measurements

**Figure 3:**
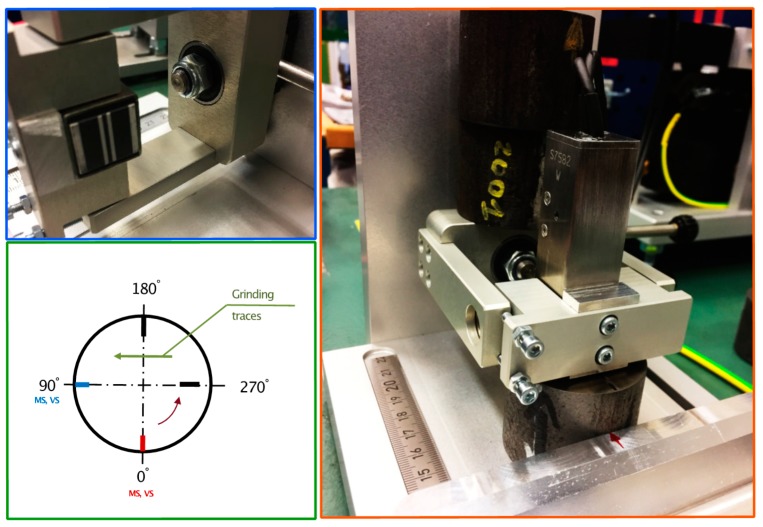
Measurement strategy for samples after grinding (MS—Microscan Software).

The Barkhausen noise analyzer Rollscan 300 (Stresstech Oy, Vaajakoski, Finland) was utilized in each laboratory with a similar type of sensor (S1-16-13-01) for flat surfaces (Figure 3). Two of the laboratories utilized the same sensor, serial number S6387, and the third laboratory utilized a sensor with the serial number S7582. The difference between the sensors was the number of coil turns. The size of both sensors was 18 × 20 mm. The measurements were carried out with Microscan software (Stresstech Oy, Vaajakoski, Finland), which records the Barkhausen noise signal and the magnetizing signal. The sweep method was utilized to determine the measurement parameters (voltage and frequency) [13]. The measurement parameters were 5 volt-peak-to-peak (Vpp) for the magnetizing voltage and 80 Hz for the magnetizing frequency. It is worth noting that the magnetic field was not of interest in this study. The bandwidth of the analyzing frequency range was 70–200 kHz. In total, 10 repetitions were carried out in two directions, referred to as the grinding direction and perpendicular to the grinding direction. The analysis was carried out perpendicular to the grinding direction, as is the standard procedure for stress and hardness change evaluation. The moving average was used for smoothing of the signal and polynomial fitting for the peak calculation. The direct results obtained from the Microscan software were utilized in the data comparison.

### 3.5. Participating Laboratories

Both industry and university laboratories in Sweden and Finland were involved in the study. The participating laboratories were as follows: Kungliga Tekniska Högskolan (KTH Royal Institute of Technology, Stockholm, Sweden) in collaboration with Scania CV AB (Södertälje, Sweden), Stresstech Oy (Vaajakoski, Finland) and Tampere University of Technology (now Tampere University, Tampere, Finland). All participating laboratories were using equipment from Stresstech Oy, Vaajakoski, Finland.

### 3.6. Datasets

Two sets of ground samples were prepared according to the full factorial design of experiments. Ten repetitions of BN measurements were carried out for each sample by the three laboratories. The measurement device calculated certain features of the BN signal. From these, the traditional RMS value together with the peak height, position, and width of the BN envelope were used. Thus, the dataset included 1440 rows of data in two separate datasets. Grinding burns were observed in two samples of dataset 1. The data from these samples were removed, as suggested in [14].

**Figure 4:**
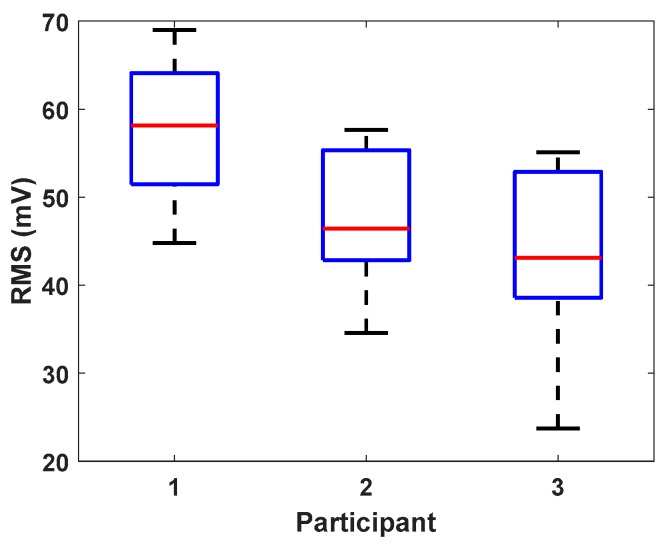
Box plot of measurement dataset 1.

One of the challenges with Barkhausen noise measurement is that the measured values may not be reproducible but depend on the measurement arrangement (participant, sensor, etc.) [15]. Figure 4 shows the box plot of dataset 1 showing that the absolute values were not reproducible. Thus, the so-called *z*-scores [16] were calculated independently for each participant with:

(2) $$z_{i}=\frac{x_{i}-x_{p}}{\hat{\sigma }}$$

where $x_{p}$ is the assigned value and $\hat{\sigma }$ is the estimated standard deviation. Both values were calculated independently for each participant. The z-scores were used in the analysis instead of the direct measurement results. The analysis of variance used the average values from 10 repetitions, while the data were more thoroughly used in the analysis of uncertainty.

### 3.7. Analysis of Variance

ANOVA is a statistical testing scheme where grouped measurements are compared with each other. The null hypothesis of the testing scheme is accepted or rejected based on the statistics calculated from the experimental data. Usually, the null hypothesis states that all the groups are random samples from the same population. The null hypothesis is rejected if the calculated p-value is lower than a predefined α-risk level. The α-risk is related to a type I error (false positive), where the null hypothesis is falsely rejected.

Depending on the data, one- or two-factor analysis can be applied. Furthermore, if the data include repeated measurements, the computational procedures differ. ANOVA employs the F-test in determining the test result. The test statistics are computed first by computing the sum of squares (SS) for the grouped measurements. By dividing the SS by its degree of freedom, the mean squares value is obtained. The mean squares of the grouped measurements are divided by the within group mean squares to come up with the F-test statistics. The within group mean squares is an approximation of the variance of the measurements under the same conditions. The computed statistics are compared with the reference value, and the p-value is computed to determine if the null hypothesis is rejected. The reference value depends on the α-risk level and the variance estimates’ degrees of freedom [16].

### 3.8. Computational Procedure

The standard ANOVA was applied to the z-scores to determine if the grinding parameters or the appraiser influenced the measured BN feature. Each experiment was repeated three times, and thus, a two-factor ANOVA with replication was applied. As mentioned above, the null hypothesis states that the grouped measurements are samples from the same population and, thus, the factor has no effect on the measured BN feature. It is rejected if the observed *F*-test statistic is greater than the reference value. The analysis of variance was carried out only for the RMS values measured. The equations for ANOVA are not presented here but can be found, for example, in [16].

**Figure 5:**
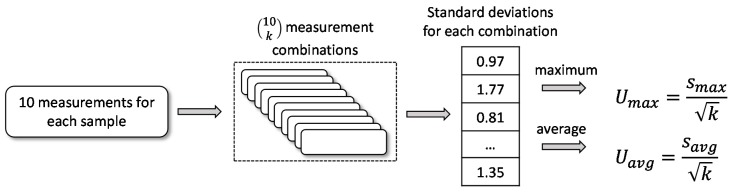
Computation of uncertainties.

Barkhausen noise is a stochastic phenomenon, and thus, only averaged properties are reproducible. Thus, the measurement needs to be repeated in order to draw conclusions reliably. The reliability involved with repeated measurements is assessed with uncertainty. In this study, uncertainty was computed to evaluate a feasible number of repetitions. Uncertainty was obtained as the standard deviation of the mean given by [14,15]:

(3) $$U=\frac{s}{\sqrt{k}}$$

where *s* is the standard deviation calculated from *p* measurements ranging from 2 to 10. However, there are $\left(\begin{array}{c} 10\\ k \end{array}\right)=\frac{10!}{k!\left(10-k\right)!}$ possible combinations of measurements depending on the value of *k*. In this study, the standard deviation for every combination was computed and followed by two uncertainty values. The first one was obtained by taking the average of $\left(\begin{array}{c} 10\\ p \end{array}\right)$ standard deviations and the second one by taking the maximum of those. The computational procedure is illustrated in Figure 5. The uncertainties were calculated for all of the features selected. The uncertainty from (3) was further divided by the average to obtain the relative uncertainty in percentages.

## 4. Results and Discussion

### 4.1. ANOVA

Table 3 shows the results of the analysis of variance for dataset 1 with the *α*-risk set to 0.05. SS is the sum of squares, df is the degree of freedom, MS is the mean squares, *F* is the *F*-test statistic, *p*-value is the probability that the observed *F*-test statistic is greater than the reference value in the case when the null hypothesis is true (i.e., the probability of falsely rejecting the null hypothesis), and *F_crit_* is the reference value for the *F*-test statistic. Table 4 shows that none of the sources of variation were statistically significant. This is quite surprising because it was assumed that the major source of variation is grinding. A careful analysis of the results given in Table 4 showed that, indeed, the variation due to grinding was the most significant, but it was not quite statistically significant at the *α*-risk set. It was also observed that the variation due to the participant was negligible. The reason for this result is that the variation within the samples was very high, indicating that the grinding had not been uniform but instead produced some unintentional changes in samples.

**Figure 6:**
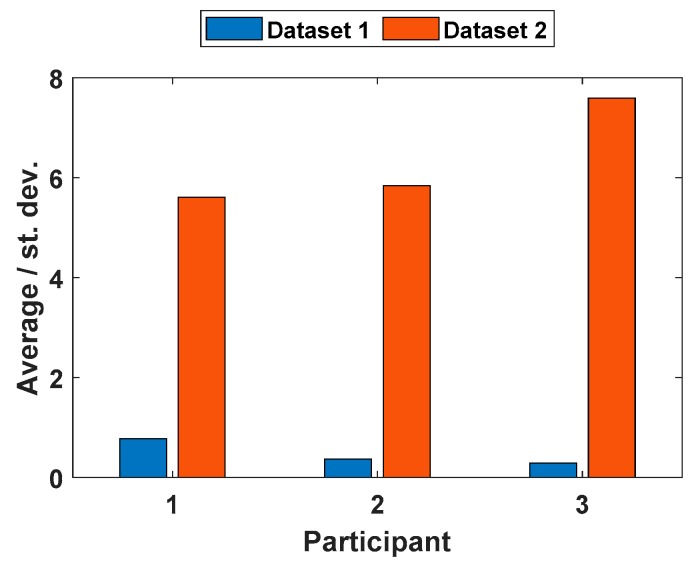
The average of the repetitions divided by the standard deviation.

To justify that the variation between the grindings with the same parameters is high in dataset 1, the average and standard deviation of the repeated experiments were computed. When the average was divided by the standard deviation, an index was obtained that could be used to evaluate the variation. Figure 6 shows the indices for both datasets and all three participants. The figure shows that for dataset 1, the index is very low, indicating that standard deviation is high compared with the average. On the other hand, the index for dataset 2 shows high values, indicating a more uniform grinding result.

Because the variation between the repeated experiments was high, a two-factor ANOVA without repetitions was carried out for dataset 1. The results are presented in Table 5. One of the conclusions is that the variation in the data was almost exclusively due to grinding parameters, while the variation due to the participant was negligible.

The results of the two-factor ANOVA with repetitions for dataset 2 are given in Table 6. This table also shows that the influence of the participant was minimal, while the major variation came from the grinding. It also shows that the interaction of the studied factors was not significant, as also indicated by Table 4.

### 4.2. Measurement Uncertainty

The measurement uncertainty calculations were carried out as described in Section 3.7. The results are presented separately for both datasets. It should be noticed that expanded standard uncertainty is usually presented where the standard uncertainty is multiplied by 2. For the results given here, the multiplication was not carried out. We used dataset 2 for calculation of the measurement uncertainty because of the higher coherence of measurement data.

**Figure 7:**
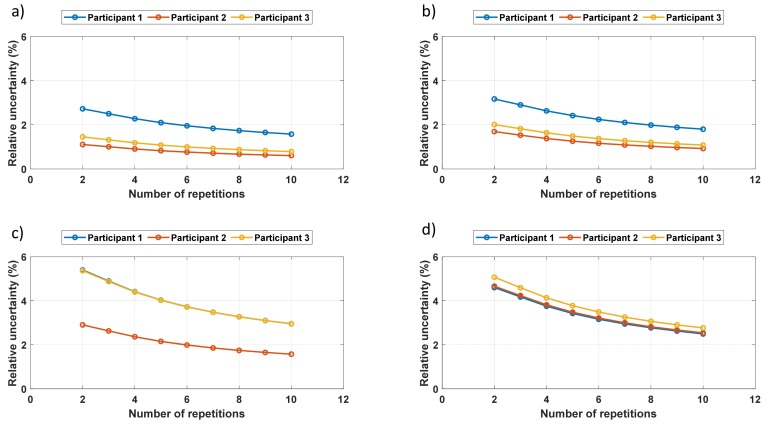
Uncertainty *U_avg_* as a function of the number of repetitions: (**a**) the RMS value, (**b**) peak height, (**c**) peak position, and (**d**) peak width.

Figure 7 shows the average uncertainties (*U_avg_*) in the features selected as a function of the number of repetitions. As expected, uncertainty decreased as the number of repetitions increased. Overall, the average uncertainties were very low, indicating that the repeated measurements by the same participant gave uniform results. One can observe that the uncertainty value depended on the participant and also on the feature used. Peak position and width had higher uncertainties, while peak height and, especially, the RMS value had lower uncertainties.

**Figure 8:**
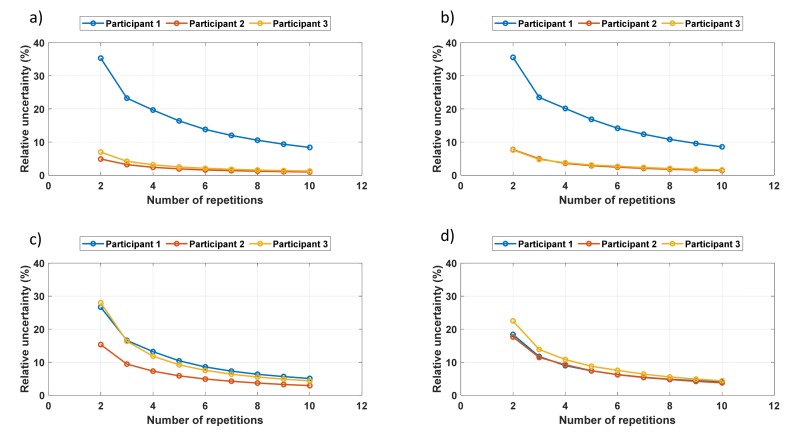
Uncertainty *U_max_* as a function of the number of repetitions: (**a**) the RMS value, (**b**) peak height, (**c**) peak position, and (**d**) peak width.

Figure 8 shows the maximum uncertainties (*U_max_*) of the features selected. The figure shows that uncertainty received high values with a small number of repetitions. The uncertainties in Figure 8 are the worst-case scenario, and thus, the values are expected to be exaggerated. Nevertheless, it still suggests that at least five repetitions were carried out. Figure 8 also shows that peak position and width had higher uncertainties than the RMS value and peak height.

The minimum set of the measurements was five, because after removing minimum and maximum values from the set, the average value could still be calculated (from the remaining three). The better solution would be to use some kind of threshold value which would give an appropriate number of measurements that need to be done. Nevertheless, Figure 7 and Figure 8 show that this is not a trivial task but a compromise between optimistic and pessimistic approaches. Therefore, we suggest making at least five measurements, but there is no upper limit for that.

### 4.3. Reproducibility of Measurement Results

Even though the presented results showed that the participant did not have a significant effect on the results, it should be noticed that the analysis was carried out with z-scores. Reading at the absolute values, it can be noticed that they were not reproducible (Figure 4). Table 7 shows the average deviations between the participants’ results. This table also shows that the values obtained by participant 1 were about 10 units higher compared with participants 2 and 3. This was probably due to the different sensor used by participant 1.

Differences may also occur in the sensor reading’s sensitivity to a measured quantity. In this study, the differences in these sensitivities were analyzed by fitting a trend line between the results of different participants. The slopes of these lines then indicated different sensitivities. Table 7 shows the equations of the trend lines. In a perfectly matching set of measurements, the slope equals 1 and the intercept equals 0. Table 8 shows that the measurements for the different datasets behaved quite differently. For dataset 2, the slopes were close to 1, indicating that the changes in the sensor readings had almost equal sensitivity to the measured quantity. However, the slopes for dataset 1 show that the results obtained by participant 3 had different sensitivity compared with participants 1 and 2.

**Figure 9:**
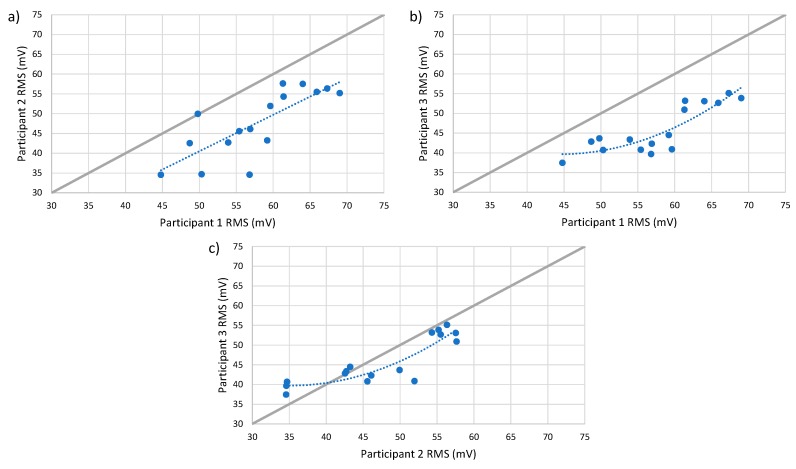
The relationship between (**a**) participants 1 and 2, (**b**) participants 1 and 3, and (**c**) participants 2 and 3 for dataset 1.

Furthermore, it was noticed that along with a different sensitivity, the relationship seems to be slightly nonlinear when participant 3 is compared with other participants in dataset 1. This is illustrated in Figure 9, which shows the scatter plots of the RMS values recorded by the participants. A polynomial trend line was also added to the figure to highlight the nonlinear relationship.

To study the influence of different grinding parameters, the average deviations between grouped measurements were computed. The groups represent measurements carried out at high and low levels of the studied variable. The average deviations are given in Table 8, where the rows are not comparable but the columns are. The Table 9 shows that hardness (i.e., tempering) was the main factor influencing the outcome of grinding. Regarding the grinding parameters, intensity had more influence on the residual stress, while abrasives had more influence on hardness.

## 5. Conclusions

This study considered proficiency testing for magnetic Barkhausen noise measurement. Three laboratories carried out measurements on two sets of samples. The samples were ground with different grinding parameters that were changed based on the design of the experiments. The standard analysis of variance was applied to the measurement results to identify the sources of variation. The dataset recorded included 10 repetitions of each measurement. This information was used to calculate uncertainties for measurements and to further identify a suitable number of repetitions needed to guarantee the validity of the results.

Uncertainty evaluation is a key factor in any metrological system when drawing conclusions about measurement results. For the Barkhausen noise technique, this approach is not fulfilled. Therefore, the study of uncertainty shown in this paper can have a great impact on future measurement setups. The main conclusions drawn from the analyses are listed below:

- In the participating laboratories, the same equipment was tested; however, because each sensor was fabricated individually, some variations in the measurement process occurred.
- Tempering before grinding was the main factor, and for the grinding parameters, intensity was the main factor affecting residual stress, while abrasives had more influence on hardness.
- The analysis showed that the absolute measurement values were not reproducible, especially if a different sensor was applied. Still, the results were reproducible and comparable. The only exception was observed for dataset 1, where the RMS values captured by one participant showed nonlinear behavior with respect to the others. The reason for this was not identified but requires further analysis.
- The uncertainty calculations suggest that at least five repetitions of Barkhausen noise measurements need to be carried out to guarantee the validity of the results.
- Manual and semimanual measurements require careful preparation and realization in order to not cause variations in measurement values. Because there are plenty of factors that can affect measurement, the best possible way is to prepare an internal standardization for sample preparation and measurement methodology and follow it every time. That will minimize preparation errors. So far, there is no global standard for the Barkhausen technique.
- Calculated uncertainty parameters follow the standardization for interlaboratory measurement evaluation, but in future work, the Monte Carlo method should be used. This is due to many parameters having an effect on the final measurement value.

## Figures and Tables

**Table 1:** General overview of the group of parameters that can affect Barkhausen measurements.

Material	Heat Treatment	Machining	Surface Integrity	Electromagnetic Properties	Measurement
Microstructure Grain size Grain shape Crystallographic defects Chemical composition Internal discontinuities Nonmetallic inclusions	Quenching Tempering Annealing Carburizing Toughening	Magnetic holders (clutch)—remanence Number and type of machining Machining parameters	Roughness Waviness Roundness Hardness Residual stresses Scratches Microcracks Burns	Magnetic domain orientation Remanence Conductance Permeability Coercivity	Gauge type Gauge quality Surface type Surface quality Surface cleanness Temperature Magnetizing voltage Magnetizing frequency Filtering bandwidth Load force Calibration Environmental noise

**Table 2:** 20MnCrS5+A material composition.

Element	C	Si	Mn	S	P	Cr	Ni	Cn	Mo	Ti	V	W
**wt %**	0.2	0.19	1.18	0.027	0.017	1.12	0.15	0.16	0.02	0.01	0.008	0.01

**Table 3:** Grinding parameters.

Batch	Material Hardness (HRC)	Grinding Variables				
		Grinding Wheel (CBN)	Grinding Wheel Speed (m/s)	Grinding Table Speed (m/min)	Infeed Rate (mm)	Cooling Fluid Emulsion 5%
**#1**	50–63%	B126	35	8	0.01	Quakercool 370
**#2**	50–57%	B181		10		Quakercool 3640

**Table 4:** The results of two-factor ANOVA with replication for dataset 1.

Source of Variation	SS	df	MS	*F*	*p*-Value	*F_crit_*
Grinding	11.85	5.00	2.37	2.37	0.06	2.48
Participant	7.11 × 10^−15^	2.00	3.55 × 10^−15^	3.55 × 10^−15^	1.00	3.26
Interaction	3.11	10.00	0.31	0.31	0.97	2.11
Within	36.04	36.00	1.00			
Total	51	53				

**Table 5:** The results of two-factor ANOVA without replication for dataset 1.

Source of Variation	SS	df	MS	*F*	*p*-Value	*F_crit_*
Grinding	39.45	17	2.32	6.83	1.11 × 10^−06^	1.93
Participant	1.42 × 10^−14^	2	7.11 × 10^−15^	2.09 × 10^−14^	1	3.28
Within	11.55	34	0.34			
Total	51	53				

**Table 6:** The results of two-factor ANOVA with replication for dataset 2.

Source of Variation	SS	df	MS	*F*	*p*-Value	*F_crit_*
Grinding	64.87	7	9.27	124.10	1.6 × 10^−28^	2.21
Participant	1.42 × 10^−14^	2	7.11 × 10^−15^	9.52 × 10^−14^	1	3.19
Interaction	0.54	14	0.04	0.52	0.91	1.90
Within	3.58	48	0.07			
Total	69	71				

**Table 7:** Average deviations between participants. Grinding burn samples removed from dataset 1.

Dataset	Participant 1 vs. 2	Participant 1 vs. 3	Participant 2 vs. 3
1	11.11	14.09	2.99
2	10.24	11.19	0.95

**Table 8:** The equations of trend lines fitted between the results of different participants. Grinding burn samples and two outlier samples were removed from dataset 1.

Dataset	Participant 1 vs. 2	Participant 1 vs. 3	Participant 2 vs. 3
1	y = 0.92x − 5.60	y = 0.72x + 4.44	y = 0.62x + 16.50
2	y = 0.95x − 6.15	y = 0.92x − 4.18	y = 0.94x + 3.14

**Table 9:** Average deviation between high and low levels tested.

Parameter	Hardness	Abrasive	Intensity
RS (MPa)	−64.46	−18.08	−45.94
FWHM	0.71	0.07	0.01
